# Lowest observed adverse effect level of pulmonary pathological alterations due to nitrous acid exposure in guinea pigs

**DOI:** 10.1186/s12199-020-00895-0

**Published:** 2020-09-26

**Authors:** Masayuki Ohyama, Hiroshi Nishimura, Kenichi Azuma, Chika Minejima, Norimichi Takenaka, Shuichi Adachi

**Affiliations:** 1 Department of Environmental Health, Osaka Institute of Public Health, 1-3-69, Nakamichi, Higashinari-ku, Osaka, 537-0025 Japan; 2 Department of Planning and Coordination, Osaka Institute of Public Health, Osaka, 537-0025 Japan; 3 Department of Environmental Medicine and Behavioural Science, Kindai University Faculty of Medicine, Osakasayama, 589-8511 Japan; 4 Department of Natural Sciences, College of Liberal Arts, International Christian University, Mitaka, 181-8585 Japan; 5 Department of Applied Chemistry, Graduate School of Engineering, Osaka Prefecture University, Sakai, 599-8531 Japan; 6 Department of Public Health, Sagami Women’s University, Sagamihara, 252-0383 Japan

**Keywords:** Nitrous acid, Nitrogen dioxide, Pulmonary emphysema, Alveolar mean linear intercept, Asthma, LOAEL

## Abstract

**Background:**

We previously demonstrated that continuous exposure to nitrous acid gas (HONO) for 4 weeks, at a concentration of 3.6 parts per million (ppm), induced pulmonary emphysema-like alterations in guinea pigs. In addition, we found that HONO affected asthma symptoms, based on the measurement of respiratory function in rats exposed to 5.8 ppm HONO. This study aimed to investigate the dose-response effects of HONO exposure on the histopathological alterations in the respiratory tract of guinea pigs to determine the lowest observed adverse effect level (LOAEL) of HONO.

**Methods:**

We continuously exposed male Hartley guinea pigs (*n* = 5) to four different concentrations of HONO (0.0, 0.1, 0.4, and 1.7 ppm) for 4 weeks (24 h/day). We performed histopathological analysis by observing lung tissue samples. We examined samples from three guinea pigs in each group under a light microscope and measured the alveolar mean linear intercept (Lm) and the thickness of the bronchial smooth muscle layer. We further examined samples from two guinea pigs in each group under a scanning electron microscope (SEM) and a transmission electron microscope (TEM).

**Results:**

We observed the following dose-dependent changes: pulmonary emphysema-like alterations in the centriacinar regions of alveolar ducts, significant increase in Lm in the 1.7 ppm HONO-exposure group, tendency for hyperplasia and pseudostratification of bronchial epithelial cells, and extension of the bronchial epithelial cells and smooth muscle cells in the alveolar duct regions.

**Conclusions:**

These histopathological findings suggest that the LOAEL of HONO is < 0.1 ppm.

## Background

Numerous epidemiological studies have shown the relationship between nitrogen dioxide (NO_2_) and the alterations of respiratory function or asthma symptoms [USEPA, 2016]. However, conventional NO_2_ assays measure nitrous acid (HONO) as NO_2_ [1]. Moreover, HONO exists in equilibrium with NO_2_, nitric oxide (NO), and water (H_2_O): 2HONO ⇆ NO + NO_2_ + H_2_O [2].

A NO_2_ threshold value of 40 μg/m^3^ (approximately 0.02 parts per million (ppm)) was set by the WHO to protect the public from the health effects of this gas. However, it is unclear to what extent the health effects observed in epidemiological studies are attributable to NO_2_ itself or to the other primary and secondary combustion-related products (such as organic carbon and HONO) to which it is typically correlated [WHO Air quality guidelines 2005 global update].

HONO may be one of the causative agents underlying the relationship between NO_2_ and asthma symptoms. A few inhalation studies have examined the association between HONO exposure and respiratory symptoms and lung function in mildly asthmatic adult subjects or in healthy adult nonsmokers [3, 4]. In a few epidemiological studies, Van Strien et al. (2004) observed that HONO exposure was not independently associated with respiratory symptoms during the first year of life [5], and Jarvis et al. (2005) observed that indoor HONO levels were associated with decreased lung function and possibly with more respiratory symptoms [6]. In an epidemiological pilot study, we observed that indoor HONO was closely correlated with outdoor NO_2_ in one research year, and indoor HONO was significantly associated with asthma attacks, according to Mann–Whitney *U* test, in another year [7].

There are various studies that have reported the HONO/NO_2_ ratio. The HONO/NOx ratio in the atmosphere at a highway junction in Houston, TX, was 1.7% [8], which was relatively high [9]. In the epidemiological study by Jarvis et al. (2005), the median indoor concentration of HONO (3.10 ppb) was approximately one-fourth that of NO_2_ (12.76 ppb), and the maximum indoor concentration of HONO (20.55 ppb) was approximately one-third that of NO_2_ (59.12 ppb) [6]. In our epidemiological pilot study, the median concentration of indoor HONO (4.70 ppb) during the weeks with mild asthma attacks was approximately 28% that of indoor NO_2_ (16.69 ppb), and approximately 36% that of outdoor NO_2_ (13.23 ppb) [7]. Incidentally, the median concentration of indoor NO during the weeks with mild asthma attacks was 4.29 ppb, and that of outdoor NO was 1.56 ppb. In addition, the median concentration of indoor HONO (2.81 ppb) during the weeks without mild asthma attacks was approximately 17% that of indoor NO_2_ (16.07 ppb), and approximately 24% that of outdoor NO_2_ (11.69 ppb). The median concentration of indoor NO during the weeks without mild asthma attacks was 5.47 ppb, and that of outdoor NO was 1.62 ppb.

We investigated the biological effects of HONO not only in an epidemiological pilot study [7] but also in animal exposure experiments [10–12]. We observed pulmonary emphysema-like alterations in the alveolar duct centriacinar regions of guinea pigs after exposure to 3.6 ppm HONO [10]; a significant increase in baseline pulmonary resistance (RLung), alveolar mean linear intercept (Lm), thickness of bronchial connective tissue near the hilar, and Muc5ac expression after 5.8 ppm HONO exposure in rats [12]; and hyperplasia of the terminal bronchial epithelial cells, with an irregular meandering and absence of dysplasia after the exposure of mice to 8.4 ppm HONO [11].

The effects of NO_2_ on RLung have not been previously reported. Sulfur dioxide (SO_2_) exposure is known to impair RLung in rats. Shore et al. (1995) reported that exposure to 250 ppm SO_2_ caused a small but significant increase in RLung and a decrease in dynamic lung compliance (Cdyn), suggesting lung fibrosis [13]. Although both NO_2_ and SO_2_ cause pulmonary emphysema-like alterations, these alterations are always accompanied by fibrosis. In contrast, HONO causes pulmonary emphysema-like alterations and an increase in baseline RLung, but not fibrosis or inflammatory changes. For example, exposure of rats to 5.8 ppm HONO did not affect baseline Cdyn or expression of chemokine (C-X-C motif) ligand 1 and tumor necrosis factor alpha [12]. Pulmonary fibrosis is rare in asthmatic patients. Therefore, HONO may impact human respiratory function more than either NO_2_ or SO_2_ alone.

We showed that pulmonary emphysema-like alterations due to HONO varied with the animal species [10–12]. After exposure to HONO, the effect of pulmonary emphysema-like alterations was observed most notably in guinea pigs [10] but not in mice [11]. Therefore, guinea pigs seemed to be the most suitable animals for observation of the histological effects of HONO.

This study aimed to investigate the dose-response effects of HONO exposure associated with histopathological alterations in the respiratory tracts of guinea pigs to find the lowest observed adverse effect level (LOAEL) of HONO. Animal exposure experiments for NO_2_ are frequently conducted at concentrations of around 20 ppm [14–16]. Therefore, based on the HONO/NO_2_ ratio obtained in the epidemiological study, our first HONO exposure experiment in animals was conducted using a HONO concentration of 3.6 ppm, and we observed clear histopathological alterations. Therefore, the HONO concentrations used in this study were established on the basis of reduction by a geometric series starting from the half concentration used in the first HONO exposure experiment.

## Methods

### Animals

Male Hartley guinea pigs (*n* = 20; body weight, approximately 360 g; age, 6 weeks) were purchased from SLC, Japan (Shizuoka, Japan). The animals were divided into four groups (*n* =5/group) and preliminarily housed for 1 week in individual animal-exposure chamber with filtered room air [10]. Briefly, the animals were raised in 0.2 m^3^ hand-made acrylic chambers with a 16 L/min flow volume of filtrated room air, using about 5 kg charcoal activated granular, 15 sheets of American air filters for vinyl isolator (Clea Japan, Inc., Tokyo, Japan), air compressors (0.4LE-8S; Hitachi Industrial Equipment Systems Co., Ltd., Tokyo, Japan), dehumidifiers (RAX3F; Orion Machinery Co., Ltd., Nagano, Japan), high-pressure regulator valves (with the largest supply pressure of about 0.078 MPa; model no. 44–2263-241; Kojima Instruments Inc., Kyoto, Japan) and mass flow controllers to control air flow (model 8350MC-0-1-1; Kojima Instruments Inc.). The internal pressure in the chambers was adjusted to about + 1 mm H_2_O relative to atmospheric pressure. Food and water were available freely during all experimental periods. The animal room was maintained under a dynamic temperature of 25 ± 2 °C to stabilize the chamber temperature and humidity. The room lighting was turned on/off by staff at 9:00 and 17:30.

### HONO exposure

In the animal-exposure chambers, the guinea pigs were exposed to filtered room air (control group: C group) or filtered room air after passing through three HONO-generation systems (low-, middle-, and high-concentration groups: L group, M group, and H group) [17]. Briefly, the HONO-generation system was based on spraying a mixture of aqueous sodium nitrite solution (> 98.5% pure sodium nitrite; Wako Pure Chemical Industries, Ltd.) and aqueous acid solution (85–92% lactic acid; Wako Pure Chemical Industries, Ltd.) in a porous polytetrafluoroethylene tube (TB-1008, approximately 15 cm length; Sumitomo Electric Fine Polymer, INC., Osaka, Japan) using filtered room air through an atomizer-nozzle (BN90s-IS[V], SUS316L, 1/8PT, M14; Atomax Co., Shizuoka, Japan). The filtered room air passing through three HONO-generation systems flowed into three HONO-exposure chambers. The aqueous solution inside the tube of HONO generation systems was discharged with a small portion of the gas. The HONO concentrations in the animal-exposure chambers were regulated based on the concentration of the aqueous sodium nitrite solution (L, M, and H groups: 2, 6, and 18 mmol/L). Guinea pigs were continuously exposed to HONO for 4 weeks. HONO exposure was stopped for approximately 3 h once every week to allow for the exchange of cages and cleaning of chambers.

### Measurement of nitrogen oxides

The HONO-measurement system has been described previously [10]. Briefly, we used a sampling method of HONO, employing two Harvard EPA Annular Denuders (URG-2000-30 × 150-3CSS; URG Corporation, NC) in the series sampling [18, 19]. The annular denuders were coated with sodium carbonate and glycerol. Air from each chamber was sampled for 30 min per day for 5 days per week with an air flow of 1 L/min, using NOx analyzer. The concentration of HONO in the air of each chamber was measured after the end of the exposure experiment, using the denuder extract with milliQ by ion chromatography (700 series; Metrohm Japan LTD., Tokyo, Japan).

The concentrations of the contaminated NO_2_ and NO were measured by a NOx analyzer (ECL-77A; J-science, Inc., Kyoto, Japan) after passing into the sodium carbonate annular denuders.

### Histopathological analysis

All animals were sacrificed after 4 weeks of HONO exposure using an overdose of pentobarbital sodium and exsanguination. For light microscopy, lung tissue samples of three guinea pigs were fixed with 10% neutral-buffered formalin (Mildform 10 NM; Wako Pure Chemical Industries, Ltd.) at 20 cm/H_2_O for the alveolar distension in each group. The lung samples were embedded in paraffin. Tissues were then sectioned and deparaffinized, stained with hematoxylin and eosin (HE) and Elastica van Gieson (EVG), and examined under a light microscope. The Lm, a measure of airspace enlargement, was examined [12]. Using light microscopy images, Lm was determined using the transparent overlay tracing of a 0.1-mm mesh hemocytometer. All intercepts with alveolar septal walls were counted at the intersection point of around five non-adjacent meshes that did not intercept bronchus or blood vessels. The total length (2 mm) of all the lines combined divided by the total number of intercepts yielded the Lm for the region studied [20, 21]. The thickness of the bronchial smooth muscle layer near the middle of the bronchus was measured using right-lung middle lobe samples. Using light microscopy, the thickness of the bronchial smooth muscle layer of each guinea pig was determined at five points around one bronchus [12].

For transmission electron microscopy (TEM) and scanning electron microscopy (SEM), lung tissue samples of two guinea pigs were fixed with 1% paraformaldehyde electron microscopy grade (TAAB Laboratories Equipment, Ltd., Berkshire, England) and 1% glutaraldehyde (20% glutaraldehyde solution; Wako Pure Chemical Industries, Ltd.) phosphate buffer at 20 cm/H_2_O for the alveolar distension in each group. The tissues were treated by routine methods and examined under TEM (JEM-1200 EX; JEOL Ltd., Tokyo, Japan) and SEM (JSM-T100; JEOL Ltd).

### Statistical analysis

Relationships between HONO-exposure concentrations and the alterations of body weight, Lm, and thickness of the bronchial smooth muscle layer were examined for statistical significance using the analysis of variance (ANOVA), followed by Dunnett’s multiple comparison. Differences associated with *p* values of < 0.05 were considered significant.

## Results

### Concentrations of nitrogen oxides and animal body weights

Table 1 shows the concentrations of HONO and secondary products of NO_2_ and NO in the animal-exposure chambers. The total sampling periods were < 2% throughout the experimental period. Although secondary products of NO were > 0.1 ppm in the M and H groups, secondary products of NO_2_ were < 0.1 ppm.

**Table 1:** Concentrations of nitrogen oxides in animal exposure chambers

Chamber	HONO	NO	NO_2_
C group	0.0	0.0	0.0
L group	0.1	0.0	0.0
M group	0.4	0.1	0.0
H group	1.7	0.2	0.0

Guinea pigs were continuously exposed to HONO for 4 weeks, the concentrations of NO_2_ and NO in each chamber air were measured for 30 min per day for 5 days per week using NOx analyser after passing into annular denuders. The concentration of HONO in each chamber air was measured using the denuder extract by ion chromatography, after the end of the exposure experiment

**Fig. 1:**
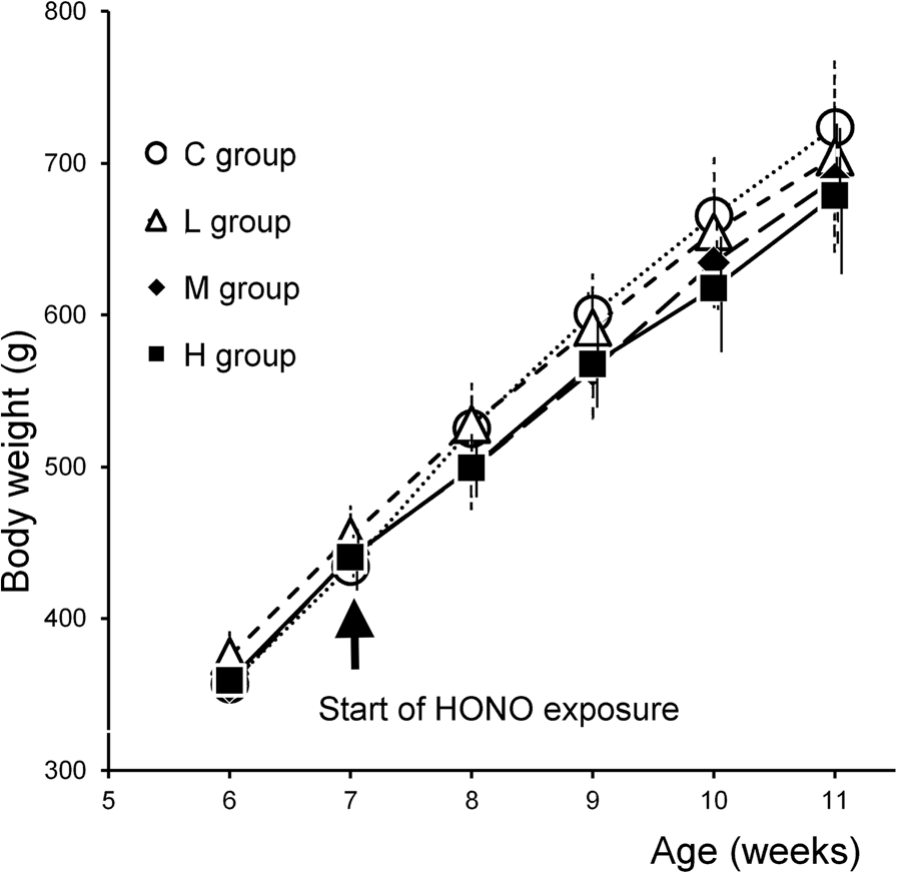
The transition of the body weight in each group. No significant differences were found by ANOVA.

Figure 1 shows the body weights of guinea pigs in each group. Although a tendency for dose-dependent decrease in body weight due to HONO exposure was observed, no significant differences were found by ANOVA.

### Effects of HONO exposure on bronchial smooth muscle and the bronchial epithelial cells

**Fig. 2:**
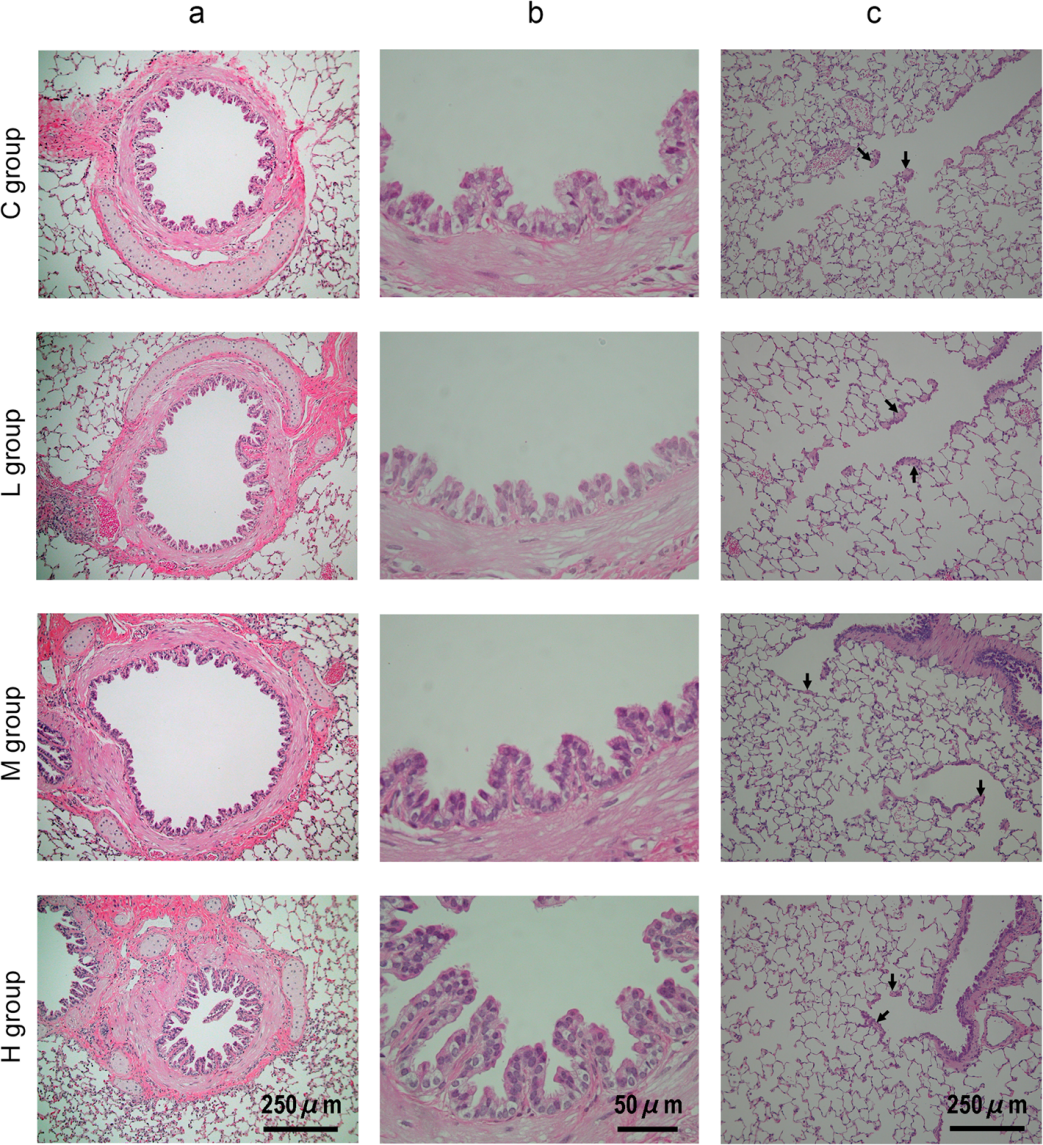
Effect of HONO exposure on the respiratory tract observed under light microscopy using hematoxylin and eosin staining. **a**, **b** Main bronchus near the middle of right-lung middle lobe. **c** Alveolar duct regions. Arrows indicate smooth muscle cells in the interstitium of the alveolar duct regions

Figure 2a shows the main bronchus near the middle of the right-lung middle lobe of guinea pigs in each group. The bronchoconstriction varied in each animal, and thus a comparison between the groups was not clear. The boundary of the smooth muscle layer in the bronchial connective tissue was clear in each group. The average thickness of the bronchial smooth muscle layer near the middle of the right-lung middle lobe is shown in Table 2. Although a tendency for thickening of bronchial smooth muscle due to HONO exposure was observed, there was no significant difference. Figure 2b shows the magnified images of Fig. 2a. There were no inflammatory alterations in any group. However, in the H group, a tendency for hyperplasia and pseudostratification of bronchial epithelial cells was observed. Figure 2c shows the alveolar duct regions of guinea pigs in each group. A dose-dependent extension of bronchial epithelial cells in the alveolar duct regions was observed, with the smooth muscle cells.

**Table 2:** Average thickness of the bronchial smooth muscle layer

Group	*n*^a^	Mean ± SD^b^	*p* value^c^	*p* value^d^
C group	3	49.6 ± 2.0	-	0.349
L group	3	53.2 ± 14.4	0.964	
M group	3	56.5 ± 12.4	0.816	
H group	3	67.5 ± 14.1	0.227	

Dunnett’s multiple comparison (one-tailed) was applied for comparison with the control, by ANOVA

^a^*n* number of animals

^b^Mean ± SD, thickness of bronchial smooth muscle layer near the middle of right-lung middle lobe

^c^*p* value, for comparison with C group

^d^*p* value, for ANOVA

### Effect of HONO exposure on pulmonary emphysema-like alterations

**Fig. 3:**
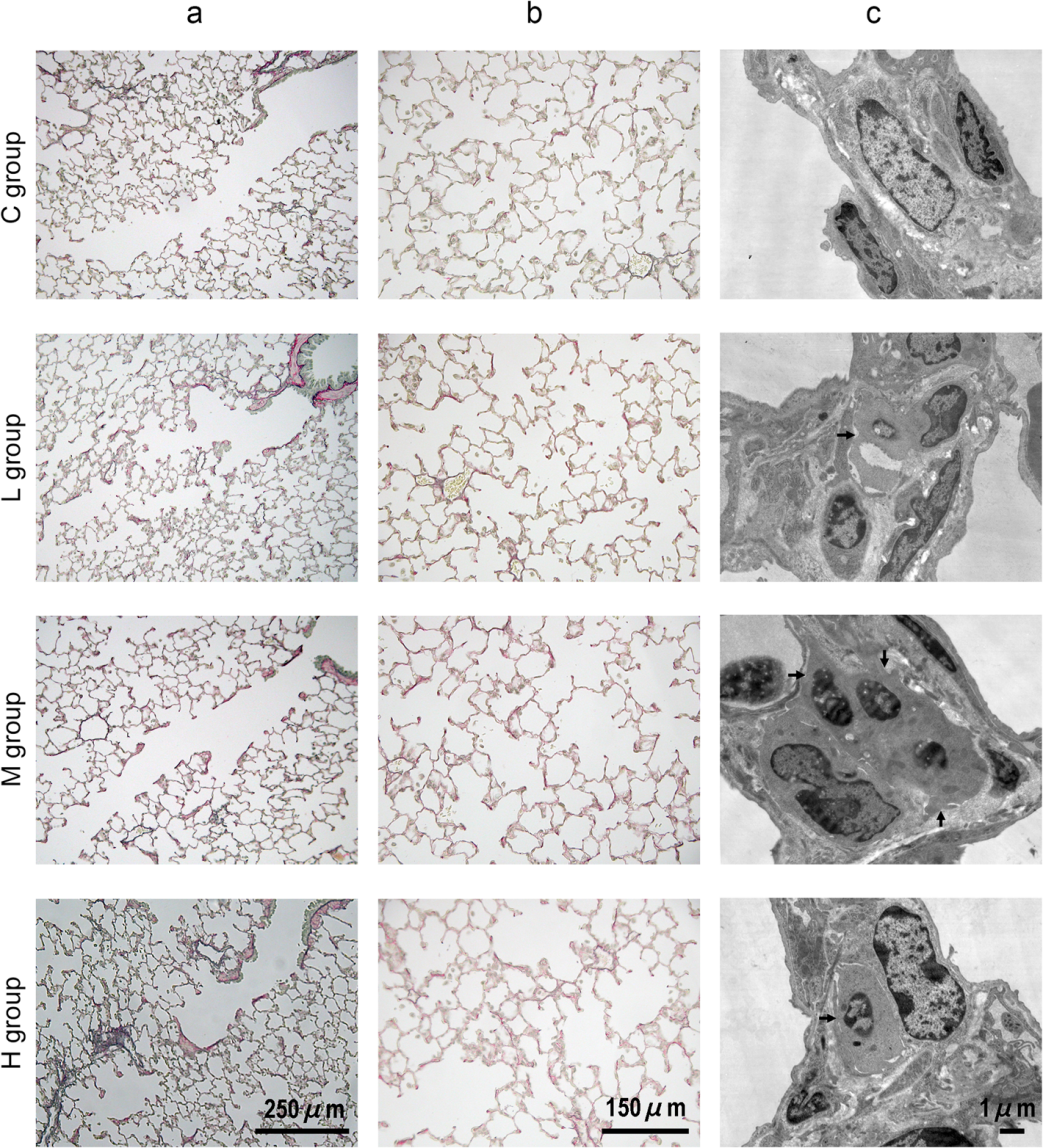
Effect of HONO exposure on the respiratory tract observed under light microscopy using Elastica van Gieson and transmission electron microscopy (TEM). **a** The terminal bronchiole and alveolar duct regions. **b** The alveolar regions. **c** Interstitium of alveolar duct regions under TEM. Arrows in the L, M, and H groups indicate smooth muscle cells that have abundant mitochondria and intermediate microfilaments

Figure 3a shows the alveolar duct regions of guinea pigs in each group. Hypertrophic alterations of the cells were observed in the alveolar duct interstitial regions in guinea pigs of the H group. A flattened surface (without fibrosis) was observed in the alveolar duct regions, and this was found to be dose-dependent. Figure 3b shows the alveoli of the guinea pigs in each group. Dose-dependent pulmonary emphysema-like alterations in the alveolar duct centriacinar regions were observed in guinea pigs of the HONO-exposure groups. Morphometric quantification of the extent of emphysema-like alterations is shown in Table 3. The average Lm of each group was 39.8–59.4 mm. The Lm of the H group was significantly longer than that of the C group according to Dunnett’s multiple comparison, but not ANOVA. Figure 3c shows the TEM images (× 5000 magnification) of the interstitium of the alveolar duct regions in guinea pigs of each group. Smooth muscle cells with abundant mitochondria and intermediate microfilaments were observed in the interstitium of the alveolar duct regions in the L, M, and H groups (consistent with the observations in Fig. 2c). The smooth muscle cells which in the interstitium of the alveolar duct regions with abundant mitochondria and intermediate microfilaments, consistent the observations in Fig. 2c. There were no edematous alterations of guinea pigs in any group.

**Table 3:** Alveolar mean linear intercept in each group after 4 weeks of HONO exposure

Group	*n*^a^	Mean ± SD^b^	*p* value^c^	*p* value^d^
C group	3	39.8 ± 2.0	-	0.081
L group	3	53.7 ± 7.1	0.146	
M group	3	51.2 ± 12.1	0.255	
H group	3	59.4 ± 7.1	0.04*	

Dunnett's multiple comparison was applied for comparison with the control by ANOVA

^a^*n* number of animals

^b^Mean ± SD, alveolar mean linear intercept

^c^*p* value, for comparison with C group (*p < 0.05)

^d^ *p* value, for ANOVA

### Effect of HONO exposure on the lung architecture

**Fig. 4:**
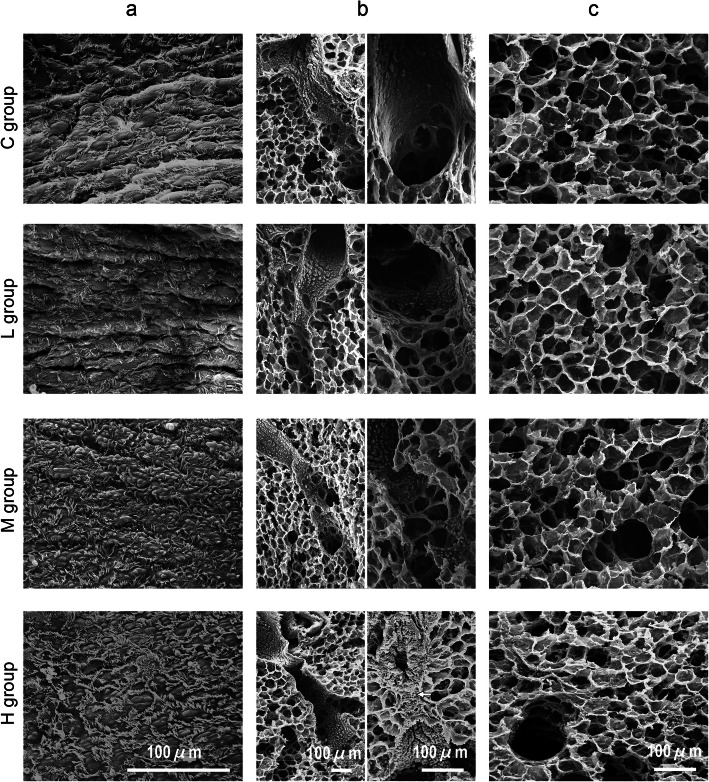
Effect of HONO exposure on the respiratory tracts, observed under scanning electron microscope. **a** The bronchial lumen near the main bronchus. **b** The alveolar duct regions. **c** The alveolar regions

Figure 4a shows the SEM images (× 500 magnification) of bronchial lumen near the main bronchus in guinea pigs of each group. The cilia of bronchial epithelial cells in guinea pigs from all the HONO-exposure groups showed no damage. A flattened surface of the bronchial epithelial cells was observed in the bronchial lumen of guinea pigs from the M and H groups, and this change was dose-dependent. Figure 4b illustrates the SEM images (at × 100 and × 200 magnifications) of the terminal bronchiole regions of guinea pigs in each group. A dose-dependent proliferative alteration of the cells was observed in the alveolar duct regions. Figure 4c demonstrates the alveolar regions of each group at × 200 magnification. Although the peripheral alveoli in the C group were normally inflated, the peripheral alveoli in the HONO-exposure groups (L, M, and H groups) were pulled in all directions and had small inflations, and this effect was dose-dependent. While the peripheral alveoli of the C group appeared to expand with low tension, the peripheral alveoli of the HONO-exposure groups (L, M, and H groups) appeared to expand with high tension. In contrast, the alveolar duct lumen of the HONO-exposure groups showed a dose-dependent expansion.

## Discussion

To the best of our knowledge, this is the first study on exposure of animals to HONO that examined pulmonary pathological alterations using SEM and TEM images. The SEM observations confirmed pulmonary emphysema-like alterations in the centriacinar regions of alveolar ducts, which were also observed under light microscopy in our previous HONO exposure experiments [10]. The SEM observations suggest that HONO exposure contracts the peripheral alveoli and expands the alveolar duct lumen. Although the pulmonary emphysema-like alterations were significant only in the H group (based on Lm measurement), alterations were also observed in the L and M groups using SEM. The TEM images showed the presence of smooth muscle cells in the interstitium of alveolar duct regions in the HONO-exposure groups and confirmed that there were no injurious effects of HONO exposure, such as edematous alterations.

In our previous HONO exposure experiments in animals, secondary products of NO_2_ and NO were sometimes present in the generated HONO, and the histopathological alterations that could be confirmed differed depending on the animal species [10–12]. This experiment had the lowest concentration of HONO in our HONO exposure experiments so far; NO_2_ (secondary product) was not detected, and pulmonary emphysema-like alterations and a tendency for hyperplasia and pseudostratification of bronchial epithelial cells were observed. In the previous HONO exposure experiments, pulmonary emphysema-like alterations were observed in guinea pigs and rats but not in mice. Although bronchial smooth muscle hypertrophy was identified in a previous HONO exposure experiment in rats, mice had few bronchial smooth muscle cells. Therefore, we speculated that HONO might induce architectural alterations in the smooth muscle cells. In contrast, the hyperplasia of the terminal bronchial epithelial cells was remarkable in mice after exposure to the highest concentration of HONO in our HONO exposure experiments so far. A tendency for hyperplasia and pseudostratification of bronchial epithelial cells was observed in this experiment. The present results suggest that these alterations were due to HONO.

In the present study, we observed pulmonary emphysema-like alterations in the centriacinar regions of alveolar ducts along with a significant increase in Lm in the H group, tendency for hyperplasia and pseudostratification of bronchial epithelial cells, and extension of bronchial epithelial cells and smooth muscle cells in alveolar duct regions. These results had a dose-dependent tendency. These histopathological results suggest that the LOAEL of HONO is < 0.1 ppm.

LOAEL is one of the important grounds of environmental quality standards (EQSs) for hazardous air pollutants. A review for EQSs with guideline values for air pollutants, including NO_2_ was reported. Although the review did not describe the LOAEL of NO_2_ from animal exposure experiments, it reported that the epidemiological effects observed in residents showed a lower value than findings from animal exposure experiments and reports on human exposure. It could be that the uncertainty factor (safety factor) was not necessary because the standards were based on data obtained from human subjects, including those with high susceptibility [22]. The EQS for NO_2_ was set as follows in 1978 in Japan: “the daily average for hourly values shall be within the 0.04–0.06 ppm zone or below that zone.” The EQS for NO_2_ is similar to the LOAEL of HONO exposure experiments in animals. The results from the present study suggest that HONO, which is detected as NO_2_, affects human health more than NO_2_ and that it is necessary to examine the involvement of HONO in the epidemiological studies of NO_2_.

The existence of HONO in the atmosphere [23] and HONO contamination in NO_2_ measurements [1] are known factors. However, the EQS for NO_2_ and the measurement method of NO_2_ have not been revised since 1978, and the WHO air quality guidelines state that it seems reasonable to retain an annual average limit for NO_2_. The reason may be that previous studies on the health effects of HONO were not sufficient for determining the EQS for HONO. Yoshida (1988) retrospectively reported the actual procedure for setting EQSs of classical pollutants, including NO_2_ [24]. In the report, the findings from animal experiments, reports on human exposure, and epidemiological studies were collected, and the dose-effect/dose-response relationship was investigated. Generally, the most important data were the epidemiological findings. There are few studies on the health effects of HONO, and two studies on human inhalation [3, 4], tree animal exposure studies [10–12], and three epidemiological studies [5–7], including our pilot study [7], have been reported. The review of EQS for NO_2_ states that scientists are in charge of proposing a guideline using scientific data, and the government is responsible for establishing its own standard. Therefore, scientists should propose a guideline for HONO, and the government should establish the EQS for HONO.

## Conclusions

We found pulmonary emphysema-like alterations in the centriacinar regions of alveolar ducts, a tendency for hyperplasia and pseudostratification of bronchial epithelial cells, and the extension of the bronchial epithelial cells and smooth muscle cells in alveolar duct regions of guinea pigs exposed to HONO. These results had a dose-dependent tendency. In our previous HONO exposure experiments in animals, secondary products of NO_2_ and NO were sometimes present in the generated HONO. However, secondary products of NO_2_ were not detected in this HONO exposure experiment. Therefore, the present results suggest that the observed alterations were caused by HONO.

## Data Availability

The datasets analyzed during the current study are available from the corresponding author on reasonable request.

## Abbreviations

Cdyn: Dynamic lung compliance
EQS: Environmental quality standard
EVG: Elastica van Gieson
HONO: Nitrous acid gas
Lm: Alveolar mean linear intercept
LOAEL: Lowest observed adverse effect level
NO_2_: Nitrogen dioxide
ppm: Parts per million
RLung: Baseline pulmonary resistance
SEM: Scanning electron microscope
SO_2_: Sulfur dioxide
TEM: Transmission electron microscope
WHO: World Health Organization

## References

[1] Pitts JN, Winer AM, Harris GW, Carter WP, Tuazon EC (1983). Trace nitrogenous species in urban atmospheres. Environ Health Perspect..

[2] Harry M, Ten B, Henk S (1998). The dark decay of HONO in environmental (smog) chambers. Atmos Environ..

[3] Beckett WS, Russi MB, Haber AD, Rivkin RM, Sullivan JR, Tameroglu Z (1995). Effect of nitrous acid on lung function in asthmatics: a chamber study. Environ Health Perspect..

[4] Rasmussen TR, Brauer M, Kjaergaard S (1995). Effects of nitrous acid exposure on human mucous membranes. Am J Respir Crit Care Med..

[5] van Strien RT, Gent JF, Belanger K, Triche E, Bracken MB, Leaderer BP (2004). Exposure to NO_2_ and nitrous acid and respiratory symptoms in the first year of life. Epidemiology.

[6] Jarvis DL, Leaderer BP, Chinn S, Burney PG (2005). Indoor nitrous acid and respiratory symptoms and lung function in adults. Thorax..

[7] Ohyama M, Nakajima T, Minejima C, Azuma K, Itano Y, Kudo S (2019). Association between indoor nitrous acid, outdoor nitrogen dioxide, and asthma attacks: results of a pilot study. Int J Environ Health Res..

[8] Rappengluck B, Lubertino G, Alvarez S, Golovko J, Czader B, Ackermann L (2013). Radical precursors and related species from traffic as observed and modeled at an urban highway junction. J. Air Waste Manage. Assoc..

[9] Trinh HT, Imanishi K, Morikawa T, Hagino H, Takenaka N (2017). Gaseous nitrous acid (HONO) and nitrogen oxides (NO x) emission from gasoline and diesel vehicles under real-world driving test cycles. J Air Waste Manag Assoc.

[10] Ohyama M, Oka K, Adachi S, Takenaka N (2010). Effects of nitrous acid exposure on pulmonary tissues in guinea pigs. Inhal Toxicol..

[11] Ohyama M, Oka K, Adachi S, Takenaka N (2011). Histological effect of nitrous acid with secondary products of nitrogen dioxide and nitric oxide exposure on pulmonary tissue in mice. J Clin Toxicol..

[12] Ohyama M, Horie I, Isohama Y, Azuma K, Adachi S, Minejima C (2018). Effects of nitrous acid exposure on baseline pulmonary resistance and Muc5ac in rats. Inhal Toxicol..

[13] Shore S, Kobzik L, Long NC, Skornik W, Van Staden CJ, Boulet L (1995). Increased airway responsiveness to inhaled methacholine in a rat model of chronic bronchitis. Am J Respir Crit Care Med..

[14] Haydon GB, Freeman G, Furiosi NJ (1965). Govert pathogenesis of NO_2_ induced emphysema in the rat. Arch Environ Health.

[15] Freeman G, Crane SC, Furiosi NJ (1969). Healing in rat lung after subacute exposure to nitrogen dioxide. Am Rev Respir Dis..

[16] Wegmann M, Fehrenbach A, Heimann S, Fehrenbach H, Renz H, Garn H (2005). NO_2_-induced airway inflammation is associated with progressive airflow limitation and development of emphysema-like lesions in C57bl/6 mice. Exp Toxicol Pathol..

[17] Ohyama M, Oka K, Adachi S, Takenaka N (2013). Development of a gaseous nitrous acid generation system for animal exposure experiments. J Clinic Toxicol..

[18] Koutrakis P, Wolfson JM, Slater JL, Brauer M, Spengler JD, Stevens RK (1988). Evaluation of an annular denuder/filter pack system to collect acidic aerosols and gases. Environ Sci Technol..

[19] Febo A, Perrino C, Cortiello M (1993). A denuder technique for the measurement of nitrous acid in urban atmospheres. Atmos Environ..

[20] Dunnill MS (1962). Quantitative methods in the study of pulmonary pathology. Thorax..

[21] Thurlbeck WM (1967). Internal surface area and other measurements in emphysema. Thorax..

[22] Kawamoto T, Pham TT, Matsuda T, Oyama T, Tanaka M, Yu HS (2011). Historical review on development of environmental quality standards and guideline values for air pollutants in Japan. Int J Hyg Environ Health..

[23] Platt U, Perner D, Harris GW, Winer AM, Pitts JN (1980). Observations of nitrous acid in an urban atmosphere by differential optical absorption. Nature..

[24] Yoshida K (1988). Ambient air quality standards. J Toxicol Sci..

